# 'Lupus headache': results from a prospective, international, inception cohort study

**DOI:** 10.1186/ar4661

**Published:** 2014-09-18

**Authors:** John G Hanly, for the Systemic Lupus International Collaborating Clinics (SLICC)

**Affiliations:** 1 Capital Health and Dalhousie University, Halifax, NS, Canada

## Background

'Lupus headache' is controversial but included in measures of global SLE disease activity. We examined the frequency, characteristics and associations of 'lupus headache' in a large, prospective, inception cohort of SLE patients.

## Methods

The study was conducted by an international network of 30 academic medical centers. Annual assessments were performed for 19 neuropsychiatric (NP) syndromes, which included five types of headache using the International Headache Society (IHS) criteria. Additional data were demographic and clinical variables, SLEDAI-2K, which includes 'lupus headache' as a standalone variable, SLICC/ACR damage index and self-report mental (MCS) and physical (PCS) component summary scores of the SF-36. Statistical analysis used linear regression models with generalized estimating equations.

## Results

Of 1,732 enrolled patients, 89% were female. Race/ethnicity was Caucasian (48%), African (16%), Asian (16%), Hispanic (16%) and other (4%). At enrollment, the mean ± SD age was 34.6 ± 13.4 years, disease duration was 5.6 ± 4.8 months and follow up was 3.8 ± 3.1 years. Twenty-six (1.5%) patients had 'lupus headache' at 27 (0.36%) of 7,523 assessments with the following IHS classification: migraine (*n *= 13), tension headaches (*n *= 8), intractable nonspecific headaches (*n *= 5), cluster headaches (*n *= 1) and intracranial hypertension (*n *= 1). In 5/27 (18.5%) assessments there were concurrent NP events. 'Lupus headache' was reported at both enrollment (*n *= 14) and follow-up (*n *= 13) assessments, in patients from all racial/ethnic groups in 15 of 30 (50%) sites located in eight of 11 countries. The estimated mean (± SE) SLEDAI-2K scores, without the 'lupus headache' variable, for visits with no headache (*n *= 6,019), a nonlupus headache (*n *= 1,330) and both a nonlupus headache and 'lupus headache' (*n *= 27) were 3.8 ± 0.08, 3.6 ± 0.18 and 7.2 ± 1.40 respectively (*P *= 0.034). Concurrent SF-36 MCS scores were 47.8 ± 0.28, 42.6 ± 0.56 and 39.4 ± 2.41 (*P *< 0.001) and PCS scores were 42.6 ± 0.30, 38.1 ± 0.53 and 32.4 ± 1.76 (*P *< 0.001). SLEDAI-2K scores, without the 'lupus headache' variable, for patients with and without 'lupus headache' were 7.2 ± 1.40 versus 3.7 ± 0.08 (*P *= 0.035). In 5/26 (19.2%) patients, 'lupus headache' was the sole contributor to the SLEDAI-2K score. Concurrent SF-36 MCS and PCS scores for patients with and without 'lupus headache' were 39.4 ± 2.41 versus 46.8 ± 0.27 (*P *= 0.002) and 32.4 ± 1.76 versus 41.7 ± 0.28 (*P *< 0.001) respectively.

## Conclusion

'Lupus headache' was infrequent, associated with higher global disease activity and a lower HRQoL. It was not reproducibly aligned with a uniform IHS classification of headache (for example, intractable headache). The lack of consistency in diagnosing 'lupus headache', even by experienced clinicians, indicates a need to better define 'lupus headache' and to reach consensus on whether it is truly a standalone manifestation of NP SLE.

